# Improved species level bacterial characterization from rhizosphere soil of wilt infected *Punica granatum*

**DOI:** 10.1038/s41598-023-35219-z

**Published:** 2023-05-27

**Authors:** Anupam J. Das, Aditya Narayan Sarangi, Renuka Ravinath, Usha Talambedu, Prasannakumar Muthukapalli Krishnareddy, Ramesh Nijalingappa, Sushil Kumar Middha

**Affiliations:** 1 School of Applied Sciences, REVA University, Bangalore, Karnataka 560064 India; 2 Basesolve Informatics Pvt. Ltd., Ellisbridge, Ahmedabad, Gujarat 380006 India; 3 Department of Plant Pathology, University of Agriculture Sciences, Bangalore, Karnataka 560065 India; 4 Department of Biochemistry, Maharani Lakshmi Ammani College for Women, Bangalore, Karnataka 560012 India; 5 Department of Biotechnology, Maharani Lakshmi Ammanni College For Women, Bangalore, Karnataka-560012, India

**Keywords:** Computational biology and bioinformatics, Environmental sciences, Genetics, Metagenomics, Soil microbiology, Biotechnology, DNA sequencing

## Abstract

Pomegranate crops are prone to wilt complex disease, which is known to severely hamper the crop yield. There have been limited studies that have explored bacteria–plant–host associations in wilt complex disease affecting pomegranate crops. In the present study, wilt infected rhizosphere soil samples (ISI, ASI) in pomegranate were studied in comparison to a healthy control (HSC). The 16S metagenomics sequencing approach using the MinION platform was employed for screening of bacterial communities and predictive functional pathways. Altered physicochemical properties in the soil samples were recorded showing a comparatively acidic pH in the ISI (6.35) and ASI (6.63) soil samples to the HSC soil (7.66), along with higher electrical conductivity in the ISI (139.5 µS/cm), ASI soil (180 µS/cm), HSC soil sample (123.33 µS/cm). While concentration of micronutrients such as Cl and B were significantly higher in the ISI and ASI soil as compared to the HSC, Cu and Zn were significantly higher in the ASI soil. The effectiveness and accuracy of 16S metagenomics studies in identifying beneficial and pathogenic bacterial communities in multi-pathogen–host systems depend on the completeness and consistency of the available 16S rRNA sequence repositories. Enhancing these repositories could significantly improve the exploratory potential of such studies. Thus, multiple 16S rRNA data repositories (RDP, GTDB, EzBioCloud, SILVA, and GreenGenes) were benchmarked, and the findings indicated that SILVA yields the most reliable matches. Consequently, SILVA was chosen for further analysis at the species level. Relative abundance estimates of bacterial species showed variations of growth promoting bacteria, namely, *Staphylococcus epidermidis, Bacillus subtilis, Bacillus megatarium, Pseudomonas aeruginosa, Pseudomonas putida, Pseudomonas stutzeri* and *Micrococcus luteus*. Functional profiling predictions employing PICRUSt2 revealed a number of enriched pathways such as transporter protein families involved in signalling and cellular processes, iron complex transport system substrate binding protein, peptidoglycan biosynthesis II (staphylococci) and TCA cycle VII (acetate-producers). In line with past reports, results suggest that an acidic pH along with the bioavailability of micronutrients such as Fe and Mn could be facilitating the prevalence and virulence of *Fusarium oxysporum,* a known causative pathogen, against the host and beneficial bacterial communities. This study identifies bacterial communities taking into account the physicochemical and other abiotic soil parameters in wilt-affected pomegranate crops. The insights obtained could be instrumental in developing effective management strategies to enhance crop yield and mitigate the impact of wilt complex disease on pomegranate crops.

## Introduction

Pomegranate (*Punica granatum*, Family: Punicaceae) is an excellent source of a variety of nutrients and minerals, dietary fibre, phenolic compounds, alkaloids and sterols. The pomegranate peel contains abundant polyphenols such as ellagitannins, gallotannins, proanthocyanidins, anthocyanins, and ellagic acid derivatives, while the seed oil is composed of unsaturated fatty acids, notably omega 5 punicic acid^1,2^. Due to the presence of these constituents the pomegranate extract and its juice have been extensively studied for their nutritive value, medicinal properties and prebiotic effects^3^. With about 2.62 lakh hectares of land dedicated to pomegranate cultivation and a production yield of 30.34 lakh tonnes, India currently holds the largest share of the global pomegranate market, accounting for over 50% of the total global production. In addition, the cultivation of pomegranate provides a source of livelihood to over 2.5 lakh families in India (NRCP Annual Report, 2020). Although persistent efforts are being made in India to improve, promote and market the crop, various factors such as abiotic and biotic stressors, physiological limitations, genetic constraints, excessive growth rate, nutrient unavailability, as well as pest and disease infestations, have been identified as severe impediments to its growth^4,5^. Diseases such as wilt complex^6^, anthracnose^7^, bacterial blight^8^, Coniella fruit rot^9,10^, foliar diseases such as leaf spot and fruit spot disease^11^ are some of the major diseases that affect the pomegranate crop. For decades, Wilt disease in pomegranate from various parts of India has reported heavy crop loss^12^. Despite the fact that bacterial-host associations and their adaptations are complex, early and accurate detection of pathogens could prevent further crop and yield loss.

16S rRNA gene sequencing has proven to be an excellent approach for identifying bacterial pathogens with higher accuracy as there are signature specific sequences in bacterial species*.* The MinION platform (Oxford Nanopore Technologies Ltd. MinION) offers a unique possibility to perform soil microbial characterization. While the use of this platform has been established in major epidemiological, laboratory-based experiments, community studies or even samples collected from remote microbiomes such as glaciers^13^, building-dust^14^, fresh water monitoring^15^, environmental metagenomes^16^, in situ bioprospecting at desert locations^17^, International Space Station (ISS)^18^, irrigation water^19^, study of ribosomal operons^20^, ebola surveillance^21^, the use of MinION in soil studies to address plant–microbe associations is limited.

Sequenced data is subject to a number of quality checks before being analysed with reference sequenced from 16S rRNA data repositories for species identification. Currently, there are a number of publicly accessible 16S reference databases such as Ribosomal Database Project^22^ (RDP, http://rdp.cme.msu.edu/), Genome Taxonomy Database^23^ (GTDB, https://gtdb.ecogenomic.org/), SILVA database^24^ (https://www.arb-silva.de/), Greengenes 16S rRNA database^25^ (https://greengenes.secondgenome.com/), and 16S-UDb^26^. Additionally, there are a few commercial solutions like EZBioCloud^27^ (https://www.ezbiocloud.net/resources) and SmartGene (https://www.smartgene.com/services/modules/16s-microbiome). The size, scope, curation methods, and frequency of updates across these databases vary greatly, as do the types of data they contain (partial sequences vs. whole genomes vs. type strains, etc.). The success of microbiome studies relies on the completeness and consistency of the existing 16S rRNA sequence repositories^26,28^. Therefore, benchmarking of multiple databases was performed to assess their taxonomy assignment potential from phylum to genus level against the gold standard NCBI’s 16S reference database. This could in turn enhance the exploratory potential, effectiveness and accuracy in identification of the pathogens.

In the present study, 16S rRNA sequencing is implemented using the MinION platform to screen for bacterial communities from wilt affected pomegranate rhizosphere soil samples. An improved approach benchmarking various 16S rRNA databases has been performed in this study. It is observed that this approach could enhance the accuracy of detection significantly minimizing false positives and negatives. Using the approach, variations in abundance of growth promoting bacteria are observed along with predicted enriched pathways. The study's findings have important implications for agriculture and crop management, and it can be inferred that identifying and promoting growth-promoting bacterial communities could be an effective strategy for improving crop yield and combating diseases. By providing insights into the microbial communities present in pomegranate crops and their potential roles in promoting growth and preventing wilt disease, the study could inform the development of targeted treatment strategies.

## Materials and methods

### Site description, sampling and physicochemical characterization

Rhizosphere soil samples were collected from an orchard close to Chikkaballapur region of Karnataka, India with coordinates of 13.3907° N, 77.6880° E. The farmer had experienced a streak of losses for five consecutive years at the time of this study, with no sign of abatement, and the losses appeared to be escalating. The soil samples were processed and the wilt infected samples were physically examined for disease symptoms confirming the presence of wilt like symptoms.

**Figure 1:**
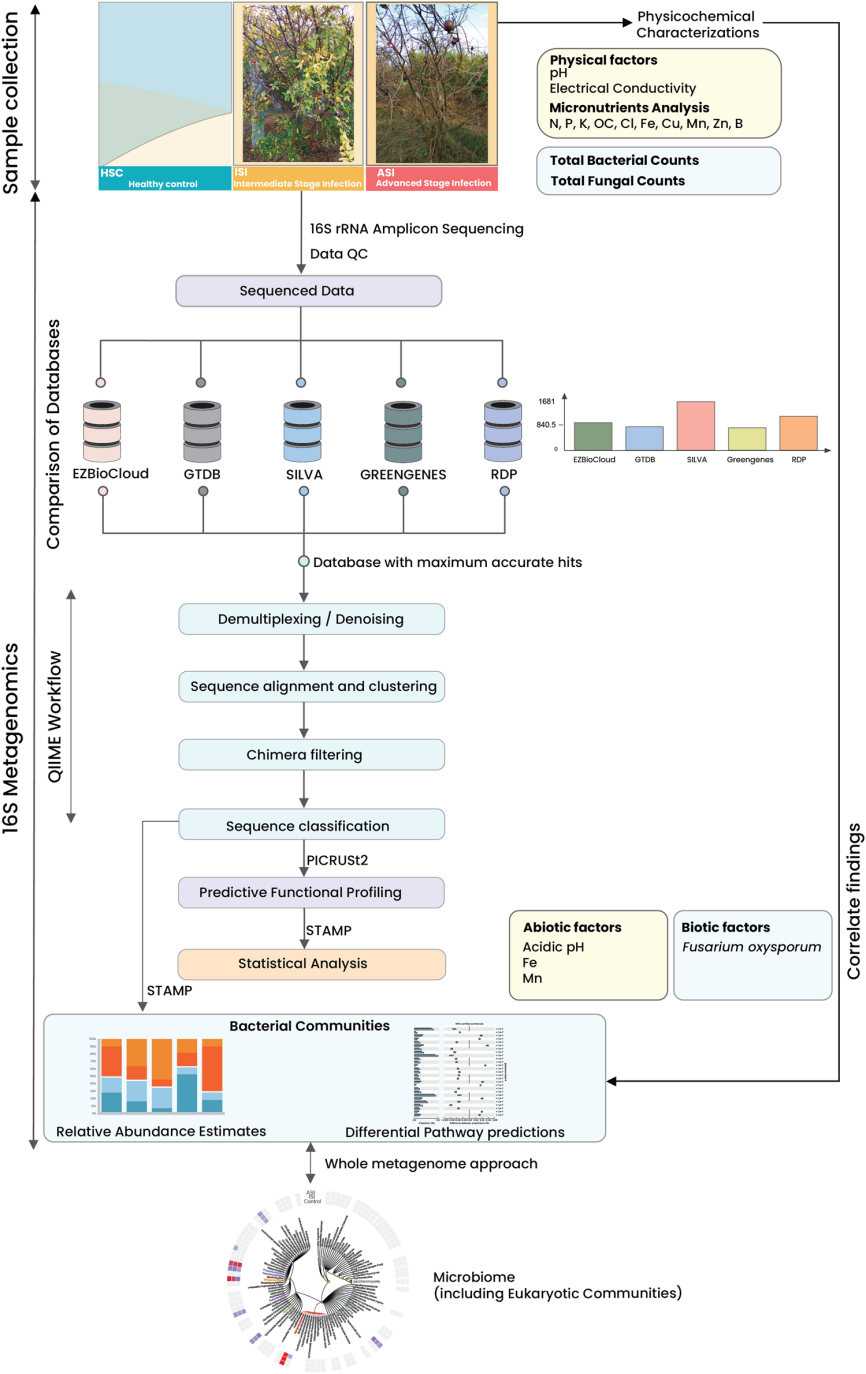
Overview of the study involving comparison of the 16S rRNA Databases and workflow.

The plants were identified as wilt infected with Intermediate Stage Infection (ISI) and Advanced Stage Infection (ASI) on the basis of physical examination of the leaves, stem, fruits and roots. In the ISI sample, the fruits had dark coloured irregular spots with cracking, whereas in the severely infected plants the fruits were completely dry with dark brown pigmentation. Leaves showed yellowing, presence of moisture, dark-coloured irregular spots in the infected plants, and complete defoliation in the ASI or severely infected samples. The root systems of the infected plants were dry and reduced with elongated galls. Dark brown colouration of the stem which had turned completely dry was observed. Severely infected plants resulted in the production of infected fruits with no recovery. Soil samples of ISI and ASI were collected from four corners and one from the center of the orchard, each taken from plants showing similar symptoms. The samples were collected in triplicates, and then pooled. The samples were submitted under the BioProject name PRJNA540763 with the accession numbers infected sample ISI (SAMN11555162; SRR9002407) and severely infected sample ASI (SAMN11555163; SRR9002406). As a control HSC, sequence data of a healthy plant sample was used from a separate study (BioProject PRJNA540834; SRR9003394). The sample was collected from the same orchard under identical conditions^29^. Whole metagenome analysis of the samples ISI and ASI has been performed and published in a separate study and the presence of *Fusarium oxysporum* has been ascertained followed by further assessment of its adaptations^30^. All the necessary permissions to carry out this study have been obtained in accordance with the local state regulations. An overview of the entire protocol is depicted in Fig. 1.

Physicochemical characterization and total microbial count estimation of the samples were carried out similar to the protocol outlined in our previous study employing whole metagenomics^30^.

### Sample preparation, microbial community DNA extraction and sequencing

#### DNA extraction and quality control

DNA from the soil samples was extracted using the commercially available DNeasy Powersoil kit (Catalog No. 12888-50) as per the manufacturer's recommendations. The soil sample was first prepared by adding it to the powerbead tube, where C1 solution was added and vortexed. Soil sample preparation was followed by cell lysis, wherein C2 solution was added and the sample was incubated at 2–8 °C. Inhibitors were removed at this stage by adding solution C3 and incubated at 2–8 °C. Binding of DNA was carried out by adding solution C4 in the MB spin column and washed with solution C5. Elution was performed by adding solution C6. Extracted DNA from the samples were quantified using the NanoDrop spectrophotometer (NanoDrop Technologies, Wilmington, DE, USA) and GEL Check before being taken for PCR amplification. The NanoDrop readings of 260/280 at an approximate value of 1.8 to 2 was used to determine the quality of DNA. Thereafter, the PCR Amplicon QC was performed, which included amplification of the 16S PCR product, which was then purified and subjected to GEL Check and NanoDrop QC. The NanoDrop readings of 260/280 with ~ value of 1.8 to 2 were inferred to be purified and used for further downstream processing.

#### PCR amplification of 16S gene

Composition of TAQ Master mix included a High-Fidelity DNA Polymerase, 0.5 mM dNTPs, 3.2 mM MgCl_2_, PCR Enzyme Buffer, and primers (16F: 5′ AGAGTTTGATCMTGGCTCAG 3′,16R: 5′ TACGGYTACCTTGTTACGACTT 3′). Extracted DNA (40 ng) was used for amplification along with 10 pM of each primer. The samples were subjected to 25 cycles of initial denaturation at 95 °C for 15 s, followed by annealing at 60 °C for 15 s, elongation at 72 °C for 2 min, and final extension at 72 °C for 10 min. The samples were finally kept at 4 °C.

#### Sequencing protocol

Nanopore sequencing was performed using 1 μg of DNA template, followed by end repair/dA tailing ligation of barcode adapter and barcoding PCR, end repair/dA tailing, blunt end adapter ligation. Thereafter purification was done using AMPure XP bead binding. Finally, the priming was carried and loaded on the SpotON flow cell.

### Metagenome sequence analysis

#### Preparation of databases

Bacterial 16S refseq nucleotide sequences (n = 22,423) were obtained from NCBI RefSeq Targeted Loci Project (https://www.ncbi.nlm.nih.gov/refseq/targetedloci/). The corresponding taxonomy in 7 lineage level hierarchy in Quantitative Insight Into Microbial Ecology (QIIME)^31^ compatible format was generated using the python script entrez_qiime.py. (https://github.com/bakerccm/entrez_qiime).

#### Ribosomal database project (RDP)

Unaligned bacterial 16S rRNA sequences (n = 31,96,041) from RDP project (https://rdp.cme.msu.edu/download/current_Bacteria_unaligned.fa.gz) were downloaded, and made QIIME compatible by sequentially removing all sequences containing < 1200, > 2000 or any ambiguous nucleotides (N). The filtered data set (n = 12,89,001 sequences) was subjected to clustering at 99% threshold using VSEARCH (2.21.1). The final dataset contained 167,789 sequences. In addition, a taxonomy mapping file in QIIME compatible format was created by linking RDP sequence identifiers of the representative sequences with 7-level (domain, phylum, class, order, family, genus, and species) lineage hierarchy.

#### GTDB

Sativa curated 16S sequences (gtdb-sbdi-sativa.r06rs202.fna; n = 46,126) from the GTDB database release R06-RS202 (https://gtdb.ecogenomic.org), were obtained from the *figshare* repository (https://scilifelab.figshare.com/articles/dataset/SBDI_Sativa_curated_16S_GTDB_database/14869077). The sequence and corresponding taxonomy mapping file was generated in QIIME compatible format.

#### EzBioCloud

16S rRNA sequences with their corresponding taxonomy in QIIME compatible format (n = 64,660) were obtained from EzBioCloud server.

#### SILVA

Silva 138 SSURef NR99 full-length sequences (n = 4,36,680) (https://data.qiime2.org/2020.6/common/silva-138-99-seqs.qza) and taxonomy (https://data.qiime2.org/2020.6/common/silva-138-99-tax.qza) in QIIME2 compatible format was obtained from QIIME data resource repository.

### Greengenes

Greengenes 16S OTUs (n = 2,03,452) and corresponding taxonomy were obtained from Greengenes FTP. ftp://greengenes.microbio.me/greengenes_release/gg_13_5/gg_13_8_otus.tar.gz.

### 16S unified database

16S rRNA sequences with their corresponding taxonomy in QIIME compatible format were obtained from 16sUDB *github* repository (https://github.com/sarangian/16S-UDb).

### Preparation of test dataset

Bacterial 16S refseq nucleotide sequences (n = 22,423) were obtained from NCBI RefSeq Targeted Loci Project (https://www.ncbi.nlm.nih.gov/refseq/targetedloci/). The corresponding taxonomy in 7 lineage level hierarchy in QIIME compatible format was generated using the python script entrez_qiime.py. (https://github.com/bakerccm/entrez_qiime).

### Comparison of the 16S rRNA sequence databases

The performances of the five 16S rRNA databases (Greengenes, SILVA, RDP, GTDB, EzBioCloud) in correctly classifying the Bacterial 16S RefSeq nucleotide sequences (NCBI RefSeq Targeted Loci Project; test dataset) were determined up to the genus levels. This comparison was done using the “classify-consensus-blast” utility of QIIME2 feature-classifier program with parameters—p-perc-identity 0.8—p-query-cov 0.8—p-maxaccepts 10. The resultant classification output (taxonomy.qza) of individual 16S databases (n = 5) were converted to tsv format and compared against the known taxonomy mapping file of the test dataset. Performance of each database was calculated as the proportion of correctly classified sequences in the test dataset.

#### ONT data analysis in QIIME2 framework

The FASTQ files were processed using the MetONTIIME pipeline (https://github.com/MaestSi/MetONTIIME), a framework based on QIIME2 using Silva V138 (Silva 138 SSURef NR99) database and BLAST classifier. Parameters used were [-n 32 -c blast -m 10 -q 0.8 -i 0.8]^32^. The resulting BIOM file and obtained representative sequences were subjected to downstream functional analysis.

#### Functional profiling predictions and statistical analysis

PICRUSt2 was employed for predictive functional profiling analysis^33^ and functional annotation of the sequences were based on Kyoto Encyclopedia of Genes and Genomes (KEGG) orthology (KO; www.kegg.jp/kegg/kegg1.html). Pathways significance differentiation was further analyzed using statistical tests. All statistical tests including differential abundance analysis was performed using STAMP 2.1.3 (http://kiwi.cs.dal.ca/Software/STAMP) for each of the samples. Two-sided Fisher’s exact test was used to compare samples Storey’s false discovery rate method of multiple test correction (p value ≤ 0.05) using DP at 95% confidence intervals^34^.

### Ethical approval

All the necessary permissions to carry out this study have been obtained in accordance with the state regulations.

## Results and discussion

The soil samples were collected from an orchard situated in Karnataka, India, which were categorized based on their stage of infection (ISI, ASI) and compared with a healthy sample (HSC).

Physical examination of the plants, with respect to their roots, leaves, stem and fruits, revealed the presence of root knots in the wilt infected plants, which could be attributed to the root knot nematode, *Meloidogyne.* The soil from infected samples had a significantly lower pH compared to the healthy sample. The pH of ISI and ASI samples were 6.35 and 6.63, respectively, as compared to a pH of 7.66 in the healthy rhizosphere soil sample. Electrical conductivity (EC) was estimated to be 139.5 µS/cm in ISI soil, and significantly higher in the ASI soil (180 µS/cm) as compared to HSC soil sample (123.33 µS/cm). An estimated total N (0.191%), P (0.01%), K (0.01%), organic carbon (OC)(0.85%), Cl(18%), Fe (0.93%), Cu (26.33 ppm), Mn (9.10 ppm), Zn (30.9 ppm), B (4.1 ppm) were reported for the ISI soil. Cl and B were significantly higher in the ISI soil as compared to the HSC soil. On the other hand, for the ASI sample, the estimated total N (0.20%), P (0.11%), K (0.014%), OC (0.97%), Cl (21%), Fe (0.98%), Cu (31.4 ppm), Mn (9.6 ppm), Zn (33.2 ppm) and B (4.3 ppm) were reported. Cl, Cu, Zn and B were found to significantly higher in the ASI as compared to the HSC soil. No significant variations in the total bacterial and total fungal counts could be found within the samples. The HSC soil had a total bacterial and total fungal counts of 176 cfu/g and 2249.3 cfu/g, respectively. Whereas the corresponding total bacterial and total fungal counts for ISI and ASI soil were 2240 cfu/g and 170 cfu/g, and 2126 cfu/g and 154 cfu/g, respectively (Table 1).

**Table 1:** Physicochemical parameters.

	pH	EC (µs/cm)	N (%)	P (%)	K (%)	OC (%)	Cl (%)	Fe (%)	Cu (ppm)	Mn (ppm)	Zn (ppm)	B (ppm)	Total Bacterial count/g cfu	Total fungal count g cfu
HSC	7.66	123.33	0.180	0.01	0.010	0.85	13.67	0.93	26.33	8.97	26.7	3.47	2249.3	176
ISI	6.35*	139.0	0.191	0.01	0.011	0.93	18.0*	0.93	29.4	9.10	30.9	4.1*	2240.0	170
ASI	6.63*	180.0*	0.200	0.011	0.014	0.97	21.0*	0.98	31.4*	9.60	33.2*	4.3*	2126.0	154

In this study, the sequencing data generated from Nanopore sequencing platform was used for taxonomic profiling of microbial communities based on 16S rRNA sequencing. Sequence metrics of the samples from the MinION sequencing were estimated to be 36,000 sequence counts (ISI) and 31,868 (ASI) samples (Table 2).

**Table 2:** Sequence information and statistics post QC.

Soil sample source	BioProject Id	Accession number	Sequence counts	Size (bp)	Average read length (bp)	Mean GC (%)
HSC healthy	PRJNA540834	SRR9003394	27,692	40,817,721	1474 ± 502	54 ± 3
ISI infected *	PRJNA540763	SRR9002407	36,000	52,955,935	1471 ± 464	54 ± 3
ASI severely infected*	PRJNA540763	SRR9002406	31,868	47,068,108	1477 ± 491	54 ± 3

*Present study.

### Comparison of databases

Accuracy in assigning bacterial lineages from Phylum to Genus level using classify-consensus-blast algorithm as furnished in Table 3.

**Table 3:** Database comparisons of phylum to genus level classification hits.

16S rRNA database	Taxonomy classification hits (number of hits, percentage)				
	Phylum	Class	Order	Family	Genus
Greengenes	22,276 (99.34%)	21,397 (95.42%)	13,431 (59.90%)	17,593 (78.45%)	13,034 (58.13%)
RDP	20,491 (91.38%)	20,017 (89.27%)	12,447 (55.51%)	11,826 (52.70%)	15,173 (67.67%)
SILVA	20,625 (91.98%)	17,282 (77.07%)	16,670 (74.34%)	18,230 (81.30%)	17,149 (76.48%)
GTDB	19,033 (84.88%)	17,170 (76.57%)	11,805 (52.65%)	14,597 (66.70%)	13,370 (59.63%)
EzBioCloud	22,290 (95.17%)	21,832 (97.36%)	19,133 (85.35%)	18,772 (83.72%)	15,229 (67.91%)

At the phylum level, the GreenGenes database revealed the maximum number of hits 22,276 (99.34%), followed by EzBioCloud 22,290 (95.17%), SILVA 20,625 (91.98%), RDP 20,491 (91.38%) and GTDB 19,033 (84.88%). The results from the comparative assessment revealed SILVA returned the maximum number of correct hits at the genus level, which was 17,149 (76.48%). EzBioCloud returned 15,229 (67.91%), followed by RDP 15,173 (67.67%), GTDB 13,370 (59.63%) and GreenGenes 13,034 (58.13%) (Table 3).

**Figure 2:**
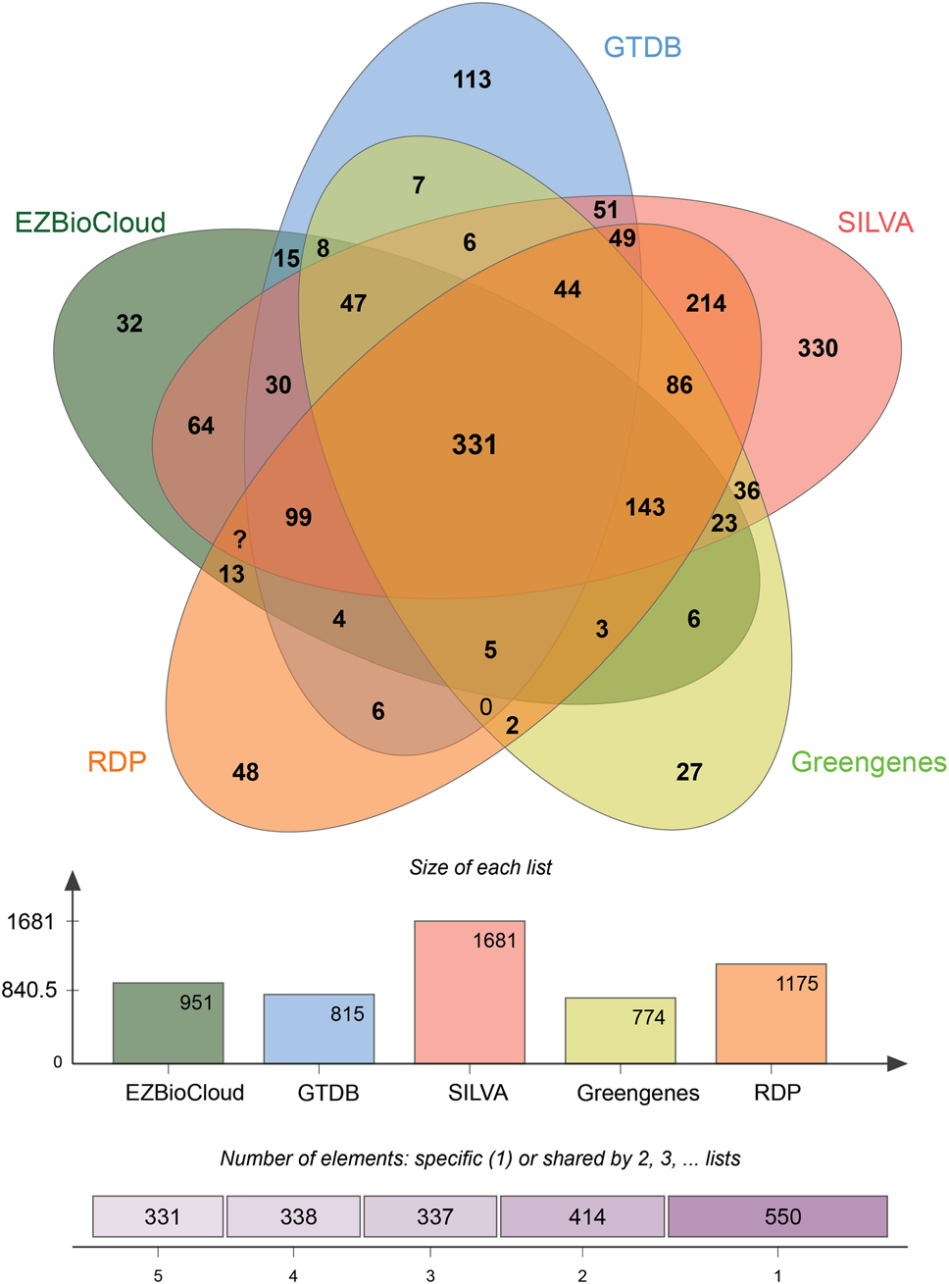
Venn representation of the results obtained from the comparison of the databases. The results from comparison of 5 popular 16s rRNA databases—EZBioCloud, GTDB, SILVA, GreenGenes and RDP.

Out of the 22,423 sequences in the test dataset, 17,149 sequences (76.48%) were correctly classified at genus level based on the SILVA database. The unique list of genera correctly identified by each of the database were subjected to JVenn for the generation of the Venn diagram (Fig. 2).

The highest number of correctly identified unique genera were returned from the SILVA database (n = 1681). Based on these results, the SILVA database was selected for further species level analysis of the samples.

#### Relative abundance estimates

**Figure 3:**
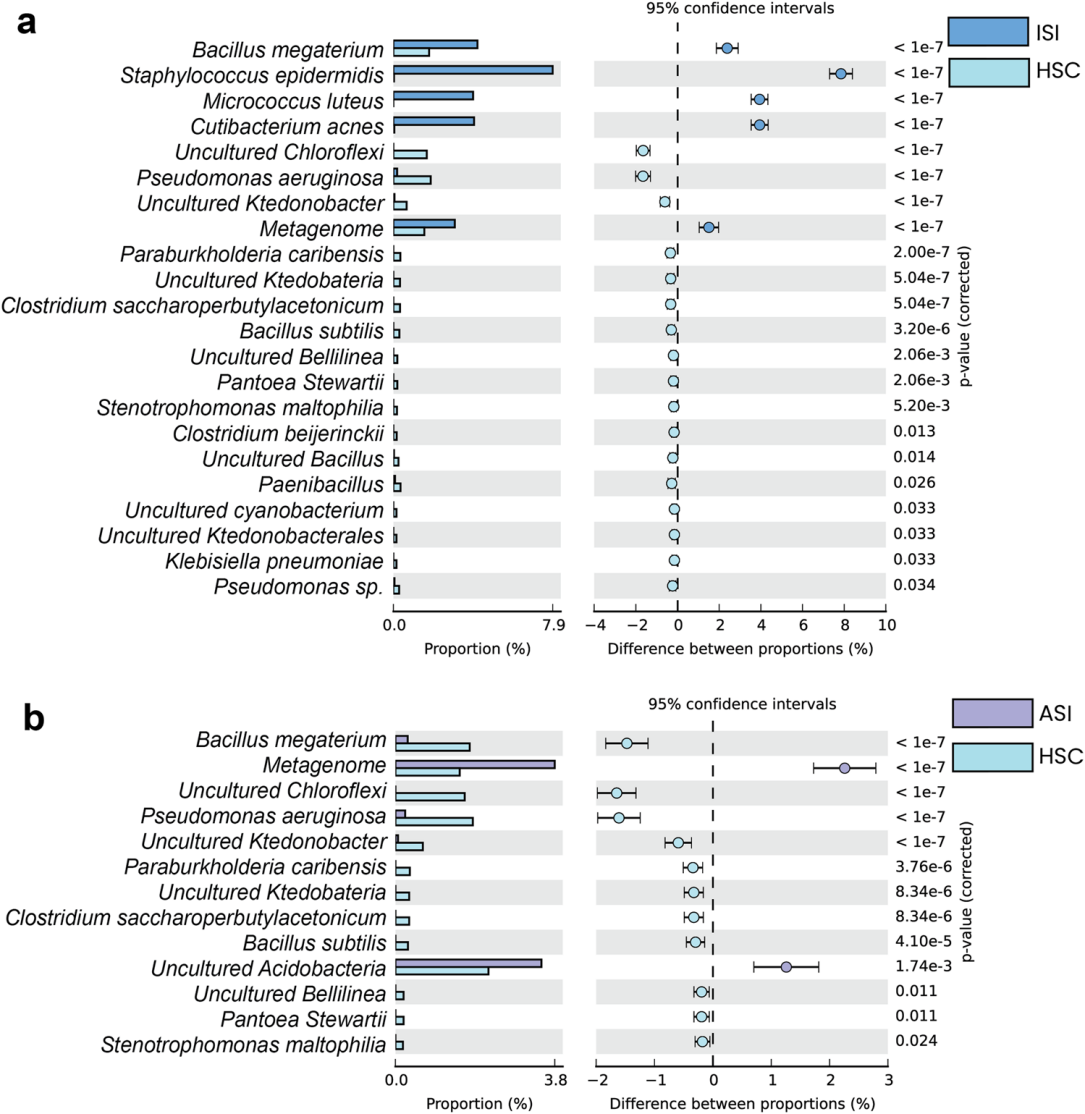
Predicted taxonomic abundance of the samples at species level. The figure depicts relative abundance of the samples at species level (**a**) relative abundance of samples ISI Vs HSC (control sample); (**b**) relative abundance of samples ASI Vs HSC (control sample); G-test (W/Yate’s) with Fischer’s test and Bonferroni’s multiple test correction has been implemented setting the significance threshold corrected-*q* value < 0.05.

Relative abundance of bacterial species level resolution showed predominance of *Staphylococcus epidermidis*,* Bacillus megatarium*,* Cutibacterium acnes*,* Micrococcus luteus*,* Pseudomonas aeruginosa*,* Pseudomonas putida*,* Pseudomonas stutzeri* in the ISI sample (Fig. 3; Table 4).

**Table 4:** Relative abundance estimates.

OTU ID	ISI	HSC	p values (corrected)	Effect size	CI	
					95% lower	95% upper
*Bacillus megaterium*	424	119	0	2.39	1.86	2.91
*Bacillus subtilis*	0	20	0	− 0.30	− 0.45	− 0.14
*Clostridium beijerinckii*	0	11	0.013	− 0.16	− 0.28	− 0.04
*Clostridium saccharoperbutylacetonicum*	0	22	0	− 0.33	− 0.49	− 0.17
*Cutibacterium acnes*	407	3	0	3.94	3.54	4.35
*Klebsiella pneumoniae*	0	10	0.03	− 0.15	− 0.26	− 0.03
Metagenome	310	103	0	1.51	1.04	1.98
*Micrococcus luteus*	402	0	0	3.94	3.54	4.34
Paenibacillus_sp.	8	24	0.02	− 0.28	− 0.45	− 0.10
*Pantoea_stewartii*	0	13	0	− 0.19	− 0.32	− 0.06
*Paraburkholderia_caribensis*	0	23	0	− 0.34	− 0.51	− 0.18
*Pseudomonas_aeruginosa*	19	124	0	− 1.66	− 2.01	− 1.30
*Pseudomonas*_sp.	5	19	0.034	− 0.23	− 0.39	− 0.08
*Staphylococcus_epidermidis*	804	2	0	7.85	7.30	8.40
*Stenotrophomonas_maltophilia*	0	12	0.005	− 0.18	− 0.30	− 0.05
s_*Bacillus*	3	17	0.014	− 0.22	− 0.37	− 0.07
Uncultured_*Bellilinea*	0	13	0.002	− 0.19	− 0.32	− 0.06
Uncultured_*Chloroflexi*	0	111	0	− 1.65	− 1.98	− 1.32
Uncultured_*cyanobacterium*	0	10	0.033	− 0.15	− 0.26	− 0.03
Uncultured_*Ktedobacteria*	0	22	0	− 0.33	− 0.49	− 0.17
Uncultured_*Ktedonobacter*	5	44	0	− 0.60	− 0.83	− 0.38
Uncultured_*Ktedonobacterales*	0	10	0.033	− 0.15	− 0.26	− 0.03

OTU ID	ASI	HSC	p values (corrected)	Effect size	CI	
					95% lower	95% upper
*Bacillus_megaterium*	24	119	0	− 1.474	− 1.836	− 1.111
*Bacillus_subtilis*	0	20	0	− 0.297	− 0.454	− 0.140
*Clostridium_saccharoperbutylacetonicum*	0	22	0	− 0.327	− 0.490	− 0.164
Metagenome	310	103	0	2.260	1.727	2.794
*Pantoea_stewartii*	0	13	0.011	− 0.193	− 0.324	− 0.062
*Paraburkholderia_caribensis*	0	23	0	− 0.342	− 0.507	− 0.176
*Pseudomonas_aeruginosa*	19	124	0	− 1.609	− 1.973	− 1.245
*Stenotrophomonas_maltophilia*	0	12	0.024	− 0.178	− 0.305	− 0.051
Uncultured_*Acidobacteria*	284	149	0.002	1.259	0.703	1.816
Uncultured_*Bellilinea*	0	13	0.011	− 0.193	− 0.324	− 0.062
Uncultured_*Chloroflexi*	0	111	0	− 1.648	− 1.979	− 1.318
Uncultured_*Ktedobacteria*	0	22	0	− 0.327	− 0.490	− 0.164
Uncultured_*Ktedonobacter*	5	44	0	− 0.592	− 0.819	− 0.366

Results show significant variations in the growth promoting bacterial species in the ISI soil sample as compared to the HSC soil. *Bacillus* species are known to produce several compounds such as antibiotics, siderophores, cell wall hydrolases and induced systemic resistance (ISR) that make them promising biocontrol agents^35,36^. *Bacillus subtilis* is a known biocontrol agent against wilt caused by C. *fimbriata*^37^*.* While significantly lower numbers of *Bacillus subtilis* were observed in the ISI soil, *Bacillus megaterium* was found to be significantly dominant in the ISI soil. *Bacillus megaterium* is known for its plant growth promoting properties and employed as biocontrol agent against pathogens such as *Alternaria japonica* and *Brassica oleracea* var*. italica*^38^*.* Furthermore, *Cutibacterium acnes*, an opportunistic human pathogen has been often reported as part of the skin flora or gastrointestinal tract. They are known to form biofilms in the skin-gland regions leading to inflammation and skin diseases. Additionally, a reduction in the number of *Pseudomonas aeruginosa* was observed. While, *Pseudomonas aeruginosa* has been reported to cause Bacterial root rot disease in Ginseng^39^, there are certain strains of *Pseudomonas aeruginosa* that are plant growth-promoting rhizobacteria (PGPR)^40^. *Staphylococcus epidermidis*, although a known human pathogen, has been reported in prior studies for their plant growth promoting properties. A study on tomato bacterial wilt disease reported the presence of endophytic bacteria *Staphylococcus epidermidis* indicating their effectiveness as biocontrol agents against *R. solanacearum*^41^*.*

On the other hand, an abundance of *Micrococcus luteus* has been reported in the ISI sample. *Micrococcus luteus* is a gram-positive bacterium that has been reported to exhibit antifungal activity^42,43^ and its growth promoting properties, biocontrol properties^44^, biotic and abiotic stress tolerance^43^. Another study has reported the growth promoting properties of *Micrococcus luteus* against *F.oxysporum* in chickpea^45^. However, there are reports of *Micrococcus luteus* causing plant diseases. A study reported the role of *Micrococcus luteus* in leafspot disease in *Mangifera indica*^46^. More experimental evidence is required to validate the role of the *Micrococcus luteus* in the pathogenesis of the wilt disease in pomegranate.

#### Predictive pathway profiling

Pathway predictions performed using PICRUSt2 and subsequently with STAMP for statistical analysis of the results revealed a significant increase in the transporter protein families involved in signalling and cellular processes (Table 5).

**Table 5:** Predictive pathway profiling.

Pathway	ISI	ASI
Metabolism	Metabolic pathways (01100) K00249	Metabolic pathways (01100) K00826, K01130, K01784, K01952
	Biosynthesis of metabolites (01110) K00249, K07024	Biosynthesis of metabolites (01110) K00826, K01952
	Fatty acid metabolism (01212) K00249	2-Oxocarboxylic acid metabolism (01210) K00826
	Carbohydrate metabolism Starch and sucrose metabolism (00500) K07024	Biosynthesis of amino acids (01230) K00826
	Lipid metabolism Fatty acid degradation (00071) K00249	Biosynthesis of nucleotide sugars (01250) K01784
	Amino acid metabolism Valine, leucine and isoleucine degradation (00280) K00249	Biosynthesis of cofactors (01240) K00826
		Carbohydrate metabolism Galactose metabolism (00052) K01784
		Amino sugar and nucleotide sugar metabolism (000520) K01784
		Lipid metabolism Sphingolipid metabolism (00600) K01130
		Nucleotide metabolism Purine metabolism (00230) K01952
		Amino acid metabolism Cysteine and methionine metabolism (00270) K00826 Valine, leucine and isoleucine degradation (00280) K00826 Valine, leucine and isoleucine biosynthesis (00290) K00826
		Glycan biosynthesis and metabolism O-Antigen nucleotide sugar biosynthesis (00541) K01784
		Metabolism of cofactors and vitamins Pantothenate and CoA biosynthesis (00770) K00826
		Biosynthesis of other secondary metabolites Glucosinolate biosynthesis (00966) K00826
Environmental information processing	Membrane transport ABC transporters (02010) K01995, K01996, K01997, K01998, K01999, K05846 Signal transduction Two-component system (02020) K03406, K07667	Signal transduction Two-component system (02020) K03406, K04771
Cellular processes	Cellular community – prokaryotes Quorum sensing (02024) K01995, K01996, K01997, K01998, K01999, K07667 Cell motility Bacterial chemotaxis (02030) K03406	Cell motility Bacterial chemotaxis (02030) K03406 Flagellar assembly (02040) K03086
Organismal systems	Endocrine system PPAR signalling pathway (03320) K00249	–
Human diseases	Endocrine and metabolic disease Alcoholic liver disease (04936) K00249	Drug resistance: antimicrobial Cationic antimicrobial peptide (CAMP) resistance (01503) K04771
Genes and proteins (Brite)		
Orthologs, modules and networks	KEGG orthology (ko00001) 27	KEGG orthology (Ko00001) 28
Protein families: metabolism	Enzymes (ko01000) K00249, K07024 Peptidases and inhibitors (ko01002) K07052	Enzymes (ko01000) K00826, K01130, K01784, K01952, K02028, K03797, K03924, K04771, K07263, K08884 Protein kinases (ko01001) K08884 Peptidases and inhibitors (ko01002) K03797, K04771, K07263 Amino acid related enzymes (ko01007) K00826
Protein families: genetic information processing	Transcription factors (ko03000) K02529	Transcription factors (ko03000) K02529
	Transcription machinery (ko03021) K03088	Transcription machinery (ko03021) K03086, K03088
		Chaperones and folding catalysts (ko03110) K04771
Protein families: signaling and cellular processes	Transporters (ko02000) K01990, K01992, K01995, K01996, K01997, K01998, K01999, K02013, K02015, K02016, K02025, K02026, K02027, K02029, K02030, K05846, K06147	Transporters (ko02000) K02004, K02015, K02025, K02026, K02027, K02028, K02029, K02030, K03559, K06147, K07114
	Secretion system (ko02044) K07667	Secretion system (ko02044) K02652, K02669
	Bacterial motility proteins (ko02035) K03406	Bacterial motility proteins (ko02035) K02652, K02669, K03406

**Figure 4:**
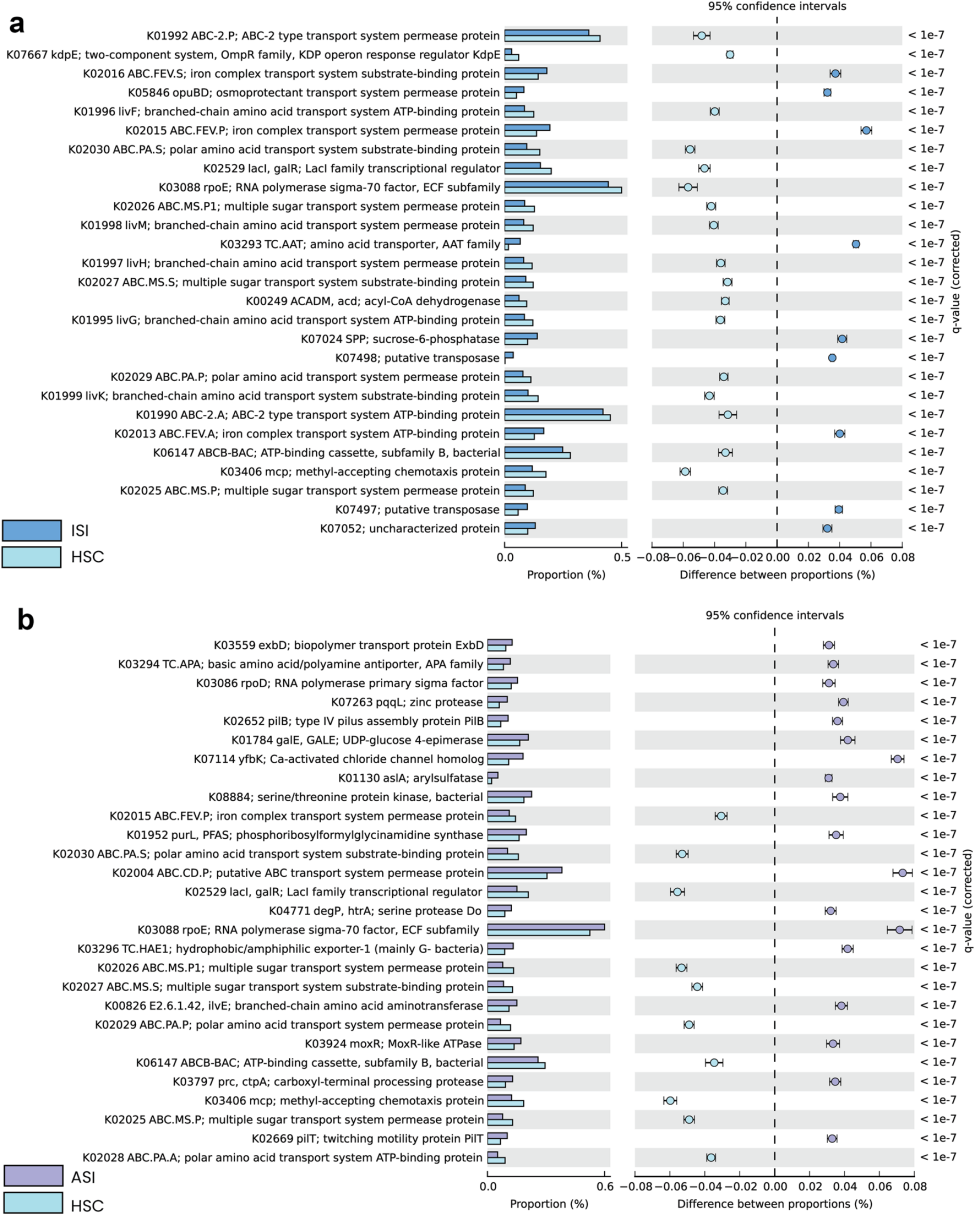
Functional predictions with KEGG Orthology matches. The figure depicts KEGG Orthology hits (**a**) KO hits ISI Vs HSC (control sample); (**b**) KO hits ASI Vs HSC (control sample);G-test (W/Yate’s) with Fischer’s test and storey FDR multiple test correction has been implemented setting the significance threshold corrected-*q*-value < 0.01 and a filter—difference between proportion with ES < 0.03.

K02015 (Iron complex transport system substrate binding protein), K02016 (Iron complex transport system substrate binding protein), K05846 osmoprotectant transport system permease protein, K03293 Amino acid transporter, K07024 sucrose-6-phosphatase and K02013 Iron-complex transport system showed significant increase in the ISI soil. The hits that showed significant increase in the ISI sample were K07498 putative transposases, K07497 putative transposase and K07052 uncharacterized protein (Fig. 4).

It is noteworthy that iron complex transport system proteins are differentially abundant in the pathways predicted in the ISI soil sample. On the other hand, the most abundant pathways predicted in comparison to the ASI soil sample were transporter proteins involved in signalling and cellular processes, K07114 Ca-activated chlorine channel (CaCC), K02004 putative ABC transport system permease protein and K03088 RNA polymerase sigma-70 factor from ECF family (Fig. 4).

**Figure 5:**
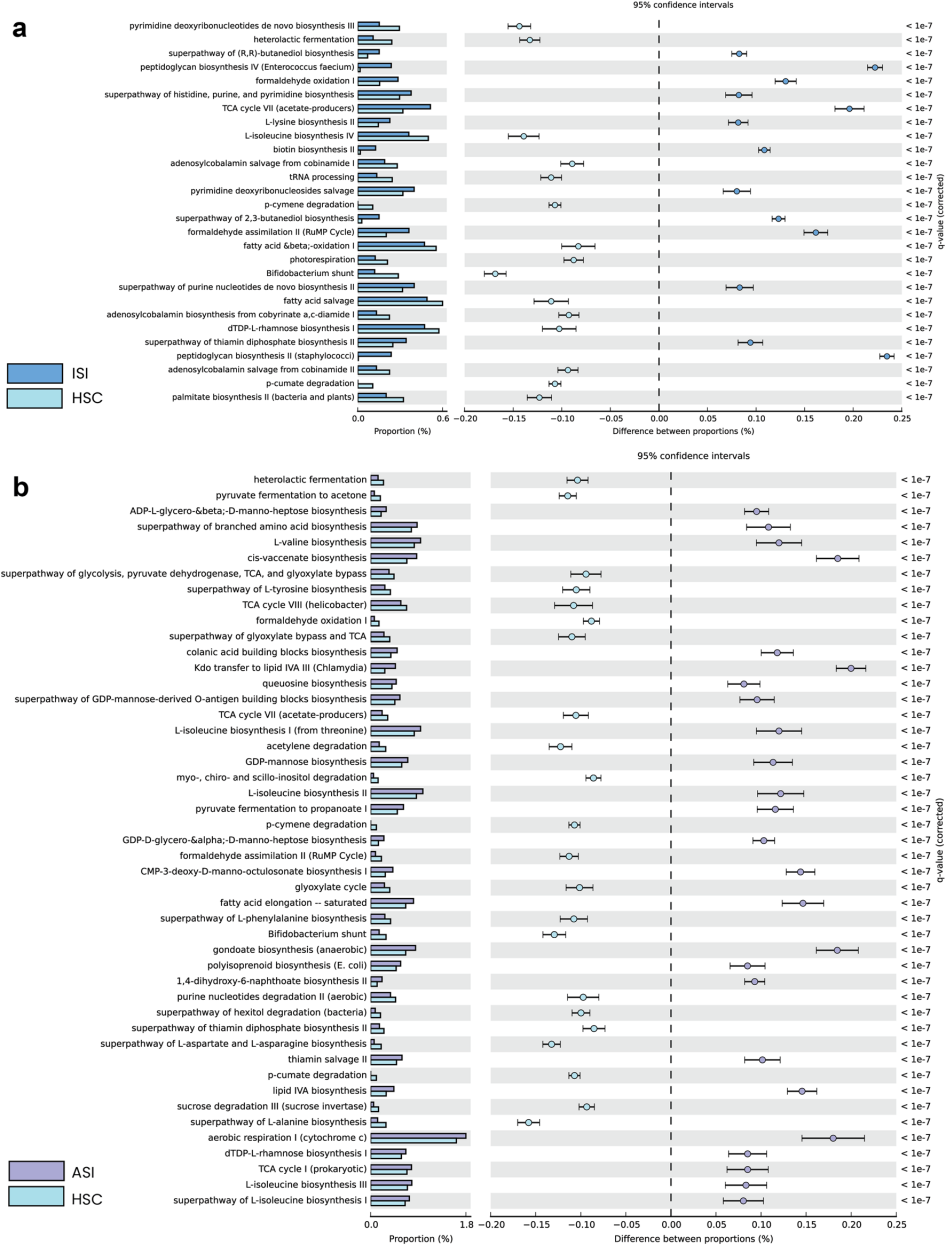
Functional predictions with Metacyc pathway matches. The figure depicts Metacyc Pathway hits (**a**) Comparison of samples ISI Vs HSC (control sample); (**b**) comparison of samples ASI Vs HSC (control sample); G-test (W/Yate’s) with Fischer’s test and storey FDR multiple test correction has been implemented setting the significance threshold corrected-*q* value < 0.01 and a filter—difference between proportion with ES < 0.08.

Peptidoglycan biosynthesis II (staphylococci) and TCA cycle VII (acetate-producers) were significantly enriched in ISI soil sample. In the ASI soil sample, aerobic respiration I (cytochrome c) and Kdo transfer to lipid IVA III (Chlamydia) pathways were found to be significantly enriched (Fig. 5).

Furthermore, correlating the findings, abiotic factors such as acidic pH, along with availability of iron (Fe) and manganese (Mn) have been reported to facilitate the growth of F. *oxysporum*, one that has a higher requirement for micronutrients^47^. The oxidation state of metals such as Fe and Mn determines their bioavailability, which is reportedly driven by the soil pH along with redox potential^48^.

The present study demonstrates the capabilities of the 16S rRNA sequencing platform in identifying potential key players involved in disease pathogenesis from soil samples collected from different pomegranate plants ranging from healthy to severely infected within the same orchard. Although various reports have described the disease symptoms in detail, the access to the diversity of the bacterial population can be facilitated through 16S rRNA sequencing using MinION. Examining the soil microbiome using 16S rRNA sequencing provides a platform for pathogenomics studies. These studies include exploring the microbial diversity and the key regulators that could provide valuable insights into the disease-causing pathogens, their adaptations and factors that influence their existence. A limitation to consider here is the amplicon-based prediction being less capable of strain-level identification. In a separate study, the microbiome of the infected soil samples the collection site have been explored extensively using the shotgun metagenomics approach^29^. This method offers improved sensitivity, resolution, and detailed characterization of microbial communities compared to traditional methods^49,50^. The study delved into the fungal communities and their adaptations, with a focus on *Fusarium oxysporum*, a known causative organism of wilt in pomegranate. The adaptations of this pathogen were also investigated. It is worth noting that wilt disease in pomegranate is caused by multiple pathogens and is often referred to as wilt complex. Furthermore, a number of beneficial bacterial communities *Staphylococcus epidermidis*,* Bacillus subtilis*,* Bacillus megatarium*, *Micrococcus luteus*,* Pseudomonas aeruginosa* were found in this study.

In particular, the present study revealed the prevalence or co-dominance of bacterial communities, which could be essential in establishing effective biocontrol strategies against wilt in pomegranate. Significant variations in the number of beneficial bacterial communities have been observed in this study. Current findings are consistent with our previous report on whole metagenome studies of infected samples^30^ and other reports from literature. The results suggest that abiotic factors, such as an acidic pH and the availability of Fe and Mn, may be contributing to the growth of *Fusarium oxysporum*, as previously observed. Past reports have recommended that limiting bioavailable micronutrients such as Fe and Mn can serve as a biocontrol strategy. However, this finding has not been validated in the present study^47^. Nonetheless, a methodology is proposed for better characterization of bacterial species through 16S metagenome analysis. Furthermore, new knowledge and significant insights into the beneficial bacterial communities and enriched pathways have been revealed that may represent functional adaptations. As mentioned earlier, the accuracy and effectiveness of 16S metagenomics studies depends on the completeness and consistency of existing 16S rRNA sequence repositories. The information derived from such repositories plays a central role in identifying key players among both beneficial and pathogenic bacterial communities, which is demonstrated in the present study by exploring the complex multi-pathogen-host systems such as the Wilt complex.

In conclusion, this study reveals the complex interactions between bacteria, soil physicochemical properties, and the wilt complex disease affecting pomegranate crops. The proposed approach has the potential to improve the utilization of 16S metagenomics sequencing data for accurate microbial identification and functional profiling predictions. Overall, the study emphasises the significance of utilizing advanced approaches and technologies to precisely detect and characterize microbial communities in agricultural settings, taking into account abiotic factors such as soil physicochemical characteristics. Further investigation could result in substantial enhancements in the management and productivity of pomegranate crops.

## Data Availability

Control (healthy) sample: https://www.ncbi.nlm.nih.gov/bioproject/?term=PRJNA540834. Infected and severely infected samples: https://www.ncbi.nlm.nih.gov/bioproject/?term=PRJNA540763. Analysis data is available at the following directory: Das et al. Supporting Data.
